# Predictors of continued HIV-risk behaviors among drug users in methadone maintenance therapy program in China—A prospective study

**DOI:** 10.1186/1477-7517-10-23

**Published:** 2013-10-10

**Authors:** Wen Chen, Yinghua Xia, Yan Hong, Brian J Hall, Li Ling

**Affiliations:** 1 Faculty of Medical Statistics and Epidemiology, School of Public Health, Sun Yat-sen University, #74, Zhongshan Road II, Guangzhou 510080, P.R China; 2 Sun Yat-sen Center for Migrant Health Policy, #74, Zhongshan Road II, Guangzhou 510080, P.R China; 3 Department of Social and Behavioral Health School of Rural Public Health Texas A&M University, TAMU 1266, College Station, TX 77843-1266, USA; 4 UNC-Project China, Guangdong STD Control Center, Guangzhou 510095, P.R China; 5 Department of Mental Health, Johns Hopkins Bloomberg School of Public Health, Hampton House, 8th Floor, 624 N. Broadway, Baltimore, MD 21205, USA

**Keywords:** Methadone maintenance therapy, HIV-risk behaviors, Longitudinal study, China

## Abstract

**Background:**

To examine the predictors of continued drug- and sex-related HIV-risk behaviors among drug users in methadone maintenance therapy (MMT) programs in China.

**Methods:**

We followed a sample of 5,035 drug users enrolled for the first time in MMT programs at baseline, 6 months, and 12 months utilizing a longitudinal prospective study design. Drug users’ HIV-risk behaviors, MMT characteristics, and drug use, were assessed at all three waves using a structured interview and HIV/HCV status was assessed at baseline and 12-month follow-up using biological specimens.

**Results:**

The point prevalence of HIV was 7.6% and 78.4% for HCV at baseline. Results of generalized linear mixed logistic regression models revealed that HIV-positive MMT clients were more likely to engage in drug injection (aOR = 1.70) and syringe sharing (aOR = 4.73). HCV-positive clients were more likely to inject drugs (aOR = 2.58), share syringes (aOR = 1.97), and have multiple sexual partners (aOR = 1.47). Adherence to MMT was the most significant predictor of reduced HIV-risk behaviors.

**Conclusions:**

Our data confirmed the positive effects of MMT on HIV prevention and underscored the urgency for programs to reduce HIV risk in HIV- and HCV-positive clients. There is a pressing need to strengthen existing counseling services for HIV-positive drug users to reduce their drug-related risk behaviors and to provide counseling for HCV-positive drug users. Further studies are needed to explore interventions to address high dropout rates and low adherence among MMT clients.

## Background

Globally, around 36 million people are addicted to heroin and opium, and many of them are HIV positive or at high risk of becoming infected and transmitting the virus to others [1]. Methadone maintenance therapy (MMT) is one of the most popular opioid replacement therapies designed to treat opioid dependence with more than one million people in MMT worldwide [2]. Despite the controversy surrounding MMT [3,4], existing literature has documented the effectiveness of MMT in reducing the risks of drug overdose, drug injection, and transmission of HIV and other blood- borne diseases [5-11]. Most of these studies were conducted in Western countries; limited data exist from developing countries like China, where more than 1.5 million drug users were recently introduced to MMT [12].

Illicit drug use reemerged in China in the early 1980s, when China adapted an open-door policy towards the West [13]. The rates of drug users continued to climb during the next decade and expanded rapidly from 70,000 in 1990 to 1.5 million in 2011 [12,13]. The Chinese government employed the same “eradication” policies instituted in the 1950s in response to the growing drug-abuse epidemic. Drug use was portrayed in the media as one of the four “social evils” subject to “fierce crack-down.” Periodic “strike-hard” campaigns were organized that targeted individuals engaging in drug use. Identified drug users were arrested and sent to detention centers for forced abstinence treatment or “reeducation through labor camps” [14]. Such policies failed to reduce the number of drug users but instead pushed them further underground. Concurrently, the first HIV epidemic broke out among injection drug users (IDU) in Yunnan in 1989 [13,15]. As of 2010, 28.6% of HIV infections in China were attributed to IDU [16].

In response to the growing HIV epidemic, the Chinese government adopted a more pragmatic approach toward drug abuse. In 2004, MMT was piloted in eight cities; MMT was scaled up nationwide since 2006 [17]. By September 2011, a total of 716 clinics across the country were approved to provide MMT and since that time, more than 332,996 drug users have been enrolled in the program [16]. The MMT system in China has thus become the largest single drug-treatment system in the world [18].

According to the national guidelines of MMT, participation in government-sponsored MMT is voluntary, and each client is required to pay 10 yuan (about $1.50) per day for the treatment. At enrollment, clients receive comprehensive medical examinations, which include testing for HIV, HCV, TB, syphilis, and liver and kidney diseases. Clients with TB and liver diseases are then referred to appropriate clinics for treatment. These examinations are performed annually during the course of MMT. If clients test positive for HIV, they are referred to the local CDC for antiretroviral treatment (ART). In addition, clients are required to take monthly urine tests for illicit drug use. Clients need to visit an MMT clinic every day. Clinicians provide them with an appropriate dose of methadone based on their individual needs. Clinicians also provide HIV prevention counseling services. A client who fails to return to a MMT clinic for 7 consecutive days is considered to have dropped out of MMT [19]. A high dropout rate has been observed in most MMT clinics in China [20-23]. The most typical reason for dropping out is detention by police or incarceration in detoxification centers. Other leading causes include trying to quit opiate use without MMT, and conflicts with job schedules [22,24].

Existing studies on MMT in China revealed that HIV-seropositive rates among IDU who participated in MMT were between 11% to 29% [10,25,26]. Studies indicate that most MMT clients continue to engage in HIV-risk behaviors while enrolled in MMT programs [10,27]. Therefore, identifying the risk factors associated with continued HIV risks is a public health priority. Available data on these risk factors come primarily from cross-sectional studies with small sample sizes [27-30]. The current study overcomes these limitations by utilizing a longitudinal prospective study design with a large sample of MMT clients. The aims of this investigation were to assess the changes in drug- and sex-related risk behaviors among MMT clients and identify the predictors of continued HIV risks.

## Methods

### Study site

Our study was conducted in Guangdong Province on the southern coast of China. Guangdong is the frontier of China’s economic reform and forerunner in terms of HIV/AIDS epidemic; the total number of HIV-infected individuals was 37, 723 in 2012 October [31] and 26.6% of HIV infections in Guangdong were transmitted through sharing injection equipment [32]. We employed a two-stage stratified sampling methodology by first choosing nine cities in Guangdong Province with different levels of economic development, and then randomly selecting one or two MMT clinics from each participating city. A total of 12 MMT clinics were selected as data collection sites for the current study representing a diverse group of 300,000 drug users [33].

### Participants

Clients were eligible to participate in our study if they met the following criteria: (a) current opioid or heroin dependence according to the International Classification of Diseases-10 (ICD-10) [34], (b) age 20 or older, (c) residence in the area/city where the MMT clinic was located (according to current Chinese policies, only residents with local household registration are eligible for local MMT services), (d) willingness to participate and provide informed consent, and (e) enrollment in the participating MMT program for the first time (to reduce the potential contamination from other HIV-prevention or drug treatment programs). At the time of the study, no other HIV prevention programs were offered in the 12 participating MMT clinics. A total of 5,624 new voluntary MMT clients meeting our criteria were enrolled in our stud. Among these participants, 319 were not tested for HIV and were excluded from the data analysis. The final sample included 5,305 participants who were followed over one year. The first follow-up at 6 months had a sample size of 3,123 (58.9%), and the second follow-up at 12 months had sample size of 2,061 (38.9%).

### Data collection

Data collection began in January 2006 and concluded in October 2010. At enrollment in the study, participants completed an informed consent and were interviewed by an experienced clinician in a private space at the MMT clinic using a structured interview questionnaire. The questionnaire consisted of demographic information and HIV-related risk behaviors designed by the National Working Group on Community-based Methadone Maintenance Treatment for Opium Dependents in China, and was widely used in MMT related studies in China [35,36]. Participants completed the same survey about their HIV- and drug-related behaviors at each follow-up and provided blood samples for HIV and HCV testing at baseline and 12-month follow-up as a part of the MMT protocol. Study participation was voluntary, and no participant received any payment or gifts for participating. The study protocol was approved by the Institutional Review Board [37] at the Sun Yat-sen University School of Public Health.

### Measurement

#### Demographic characteristics

Were collected during the baseline survey, including age, gender, education, employment status, marital status, and history of drug abuse.

#### MMT characteristics

The MMT clinicians recorded clients’ daily methadone dosage and other medical information. Client attendance was tracked and the criterion of MMT dropout (and from this current study) was missing 7 consecutive days of MMT. In addition, clients provided monthly urine samples to test for illicit drug use. From these data, we identified three treatment characteristics of the participants; first, *mean dosage of methadone*, the average amount of methadone the client had taken; second, *treatment adherence*, the number of days the client adhered to the treatment guidelines and took daily dosage of methadone as required; and third, *longest duration of abstinence (LDA)*, defined as the longest duration of negative urine tests for drug use.

#### HIV-risk behaviors

*Drug-related risk behaviors* were assessed by inquiring if the participant had injected drugs and whether he or she had shared syringes in the past one month. *Sex-related risk behaviors* included whether the participant had unprotected sex in the previous sexual intercourse or whether he or she had multiple sexual partners (more than one) in the past 3 months.

#### HIV- and HCV-positive status

MMT clients provided blood samples for HIV and HCV serum antibody testing at enrollment and 12-month follow-up in the MMT program. Samples were first screened by latex chromatographic, positive samples were further confirmed using the immunoblotting method by the local Centers of Disease Control. Meanwhile, blood samples from each client were tested for hepatitis C antibody by enzyme-linked immunosorbent assay (ELISA). Pre- and posttest counseling services were provided. National guidelines were followed in all counseling and testing services.

### Data analysis

First, we used *t*-test or Fisher’s exact test to compare the key demographics, MMT characteristics, and HIV- and HCV-infection status across the samples at baseline, first follow-up, and second follow-up as well as between the retained sample and dropout sample. Second, we identified the time trend of both drug- and sex-related HIV-risk behaviors during the course of treatment. To best utilize available data and to account for intra-class correlation (ICC) resulting from repeated measures, we used generalized linear mixed models (GLMM) for binary outcomes with a logit link function. Finally, we used logistic regression with GLMM to estimate unadjusted and adjusted odds ratio (aOR) and 95% confidence intervals (CI) for the predictors of continued HIV-risk behaviors. Four bivariate models (to produce the “unadjusted OR” for four types of HIV risk behaviors) and four multivariable models (to produce the “adjusted OR” for four types of HIV risk behaviors) were conducted on outcomes obtained at 12-month follow-up. Variance components, with the VC command, was chosen as covariance structure for the repeated measures, based on Akaike’s information criteria (AIC) [38]. All statistical analyses were performed using SAS 9.3 and the SAS procedure PROC GLIMMIX (SAS Institute Inc., Cary, NC, USA).

## Results

### Demographic and MMT characteristics over 12 months of follow-up

As depicted in Table 1, at the baseline, the mean age of MMT participants was 38.6 years (SD = 6.17), and the mean length of drug use prior to MMT was 15 years (SD = 5). Most participants were male (93%), 46% were married, 58% were employed, and 18% had completed secondary school. Approximately 7.6% were HIV positive and 78.4% were HCV positive. Participants in the first follow-up had a mean 183 treatment-adherence days and a median 4 months of abstinence. Participants in the second follow-up had a mean 267 treatment-adherence days and a median 4 months of abstinence. As shown in Table 2, the retained participants and dropouts differed significantly in most key demographic and MMT characteristics. Dropouts were more likely to be younger, male, less educated, HIV-positive, HCV-positive, with a shorter drug use history, a lower dosage of methadone, and a shorter duration of abstinence.

**Table 1:** Demographic and MMT characteristics of MMT clients during the course of treatment

**Characteristics**	**Baseline**	**6-month follow-up**	**12-month follow-up**
	**(*n*** **= 5305)**	**(*n*** **= 3123)**	**(*n*** **= 2061)**
Age (years), mean (SD)	38.65(6.17)	38.63(6.15)	38.85(6.24)
Sex *n* (%)			
Male	4916 (92.67)	2865(91.74)	1898(92.09)
Female	389(7.33)	258(8.26)	163(7.91)
Employment status *n* (%)			
Employed	3066(57.79)	1928(61.74)	1287(62.45)
Unemployed	2239(42.21)	1195(38.26)	774(37.55)
Marriage status *n* (%)			
Married	2449(46.16)	1445(46.26)	968(46.97)
Unmarried	2856(53.84)	1698(53.74)	1093(53.03)
Education *n* (%)			
Primary school	1038(19.57)	611(19.56)	406(19.70)
Junior school	3335(62.87)	1923(61.58)	1241(60.21)
Senior school or higher	932(17.57)	589(18.86)	414(20.19)
HIV *n* (%)			
Positive	401(7.56)	206(6.60)	148(7.18)
Negative	4904(92.44)	2917(93.40)	1913(92.82)
HCV *n* (%)			
Positive	3909(78.38)	2427(79.73)	1626(80.34)
Negative	1078(21.62)	617(20.27)	398(19.66)
Years of drug use before MMT mean (SD)	15.02(5.04)	15.22(5.06)	15.45(4.88)
Methadone dosage (mg/day)	--	51.11(22.05)	52.38(22.17)
mean (SD)			
Treatment adherence (days)	--	183.00	267.00
mean (SD)		(95.00 ~ 183.00)	(152.00 ~ 336.00)
Longest duration of abstinence (months) median (IQR)	--	4.00 (2.00 ~ 6.00)	4.00(2.00 ~ 7.00)

**Table 2:** Demographic and MMT characteristics between retained and dropout clients at 6- and 12- month follow-up

**Characteristic**	**6-month follow-up**		**12-month follow-up**	
	**Retained**	**Dropped out**	**Retained**	**Dropped out**
	**(*n*** **= 3123)**	**(*n*** **= 2182)**	**(*n*** **= 2061)**	**(*n*** **= 3244)**
Age (yrs) mean (SD)	38.63(6.15)	38.23(6.10) ^**^	38.85(6.24)	38.27(6.07) ^**^
Sex *n* (%)				
Male	2865 (91.74)	2071 (94.91) ^***^	1898 (92.09)	3038 (93.65) ^**^
Female	258 (8.26)	111 (5.09)	163 (7.91)	206 (6.35)
Employment *n* (%)				
Employed	1928 (61.74)	1118 (51.24) ^***^	1287 (62.45)	1759(54.22) ^***^
Unemployed	1195 (38.26)	1064 (48.76)	774 (37.55)	1485(45.78)
Marital status *n* (%)				
Married	1445 (46.26)	1004 (46.01)	968 (46.97)	1481(45.65) ^*^
Unmarried	1698 (53.74)	1178(53.99)	1093 (53.03)	1763(54.96)
Education *n* (%)				
Primary school	611(19.56)	427(19.57) ^*^	406(19.70)	632(19.48) ^***^
Junior school	1923(61.58)	1412(64.71)	1241(60.21)	2094(64.55)
Senior school or higher	589(18.86)	343(15.72)	414(20.19)	518(15.97)
HIV *n* (%)				
Positive	206(6.60)	195(8.94) ^**^	148(7.18)	253(7.80)
Negative	2917(93.40)	1987(91.06)	1913(92.82)	2991(92.20)
HCV *n* (%)				
Positive	2427(79.73)	1482(76.27) ^**^	1626(80.34)	2283(77.05) ^**^
Negative	617(20.27)	461(23.73)	398(19.66)	680(22.95)
Yrs of drug use before MMT	15.22(5.06)	14.74(5.00) ^**^	15.45(4.88)	14.75(5.12) ^***^
mean (SD)				
Methadone dosage (mg/day) mean (SD)	51.11(22.05)	44.10(18.94) ^***^	52.38(22.17)	45.59(19.98) ^***^
Treatment adherence (days), mean (SD)	183.00	36.00	267.00	53.00
	(95.00 ~ 183.00)	(14.00 ~ 90.00) ^***^	(152.00 ~ 336.00)	(18.00 ~ 143.00) ^***^
Longest duration of abstinence (months) median (IQR)	4.00(2.00 ~ 6.00)	0.00(0.00 ~ 1.00) ^***^	4.00(2.00 ~ 7.00)	1.00(0.00 ~ 2.75) ^***^

^*^p < 0.05, ^**^p < 0.01, ^***^p < 0.001.

### Changes of HIV risk behaviors at three measurements

As shown in Table 3, drug-related HIV-risk behaviors decreased throughout the course of treatment. Injecting drugs in the past month decreased from 82% at baseline to 30% at first follow-up and 22.5% at second follow-up. Sharing needles decreased from 18.6% to 4.9% to 4.0%. Sex-related risk behaviors had mixed responses to the treatment. For instance, having unprotected sex in the last sexual intercourse decreased from 56.9% to 54.1% at first follow-up and remained at the same level at second follow-up. Having multiple sexual partners in the past 3 months decreased from 9.4% at baseline to 6.8% at first follow-up but increased to 9.3% at second follow-up.

**Table 3:** Changes in HIV risk behaviors during the course of MMT treatment

**Risk behaviors**	**Baseline**	**6-month follow-up**	**12-month follow-up**
	**(*n*** **= 5305)**	**(*n*** **= 3123)**	**(*n*** **= 2061)**
Drug injection in the past 1 month			
Yes	4368(82.34)	937(30.00)	464(22.51) ^***^
No	937(17.66)	2186(70.00)	1597(77.49)
Syringe sharing in the past 1 month			
Yes	988(18.62)	154(4.93)	83(4.03) ^***^
No	4317(81.38)	2969(95.07)	1978(95.97)
Unprotected sex at last sexual encounter			
Yes	1782(56.88)	1078(54.09)	695(54.64) ^*^
No	1351(43.12)	915(45.91)	577(45.36)
Multiple sexual partners in the past 3 months			
Yes	401(9.35)	213(6.83)	118(9.30) ^***^
No	3887(90.65)	2905(93.17)	1151(90.70)

Main effect of time: ^*^p < 0.05, ^***^p < 0.001.

### Predictors of continued HIV-risk behaviors

Tables 4 and 5 present the predictors of drug- and sex-related risk behaviors. In the multivariable model, after controlling for potential confounders and ICC, the following variables significantly increased the risk of drug injection: younger age, longer history of drug use, being male, unemployment, not completing secondary school, being unmarried, HIV positive, HCV positive, lower mean dosage of methadone, lower level of treatment adherence and shorter duration of abstinence.

**Table 4:** Generalized linear mixed models (GLMM) logistic regression results: Predictors of continued drug-related risk behaviors

**Variables**	**Drug injection**		**Syringe sharing**	
	**Unadjusted OR**	**Adjusted OR**	**Unadjusted OR**	**Adjusted OR**
Age	0.99 (0.98 ~ 0.99) ^***^	0.98(0.97 ~ 0.99) ^***^	0.97(0.96 ~ 0.98) ^***^	0.97(0.95 ~ 0.98)^***^
Years of drug use before MMT	1.03(1.02 ~ 1.04) ^***^	1.04(1.03 ~ 1.06) ^***^	1.03(1.02 ~ 1.05) ^***^	1.03(1.01 ~ 1.05)^**^
Sex				
Male	1.55(1.35 ~ 1.79) ^***^	1.35(1.08 ~ 1.69) ^*^	1.62(1.25 ~ 2.10) ^***^	1.27(0.90 ~ 1.79)
Female	Reference	Reference	Reference	Reference
Employment status				
Employed	0.96 (0.89 ~ 1.04)	0.85(0.74 ~ 0.97) ^*^	1.04(0.92 ~ 1.17)	1.02(0.85 ~ 1.23)
Unemployed	Reference	Reference	Reference	Reference
Education				
Senior school or higher	0.69(0.61 ~ 0.78) ^***^	0.71(0.58 ~ 0.88) ^**^	0.61(0.49 ~ 0.75) ^***^	0.81(0.59 ~ 1.12)
Junior school	1.00(0.91 ~ 1.11)	0.98(0.83 ~ 1.16)	1.03(0.23 ~ 4.67) ^***^	1.19(0.95 ~ 1.49)
Primary school	Reference	Reference	Reference	Reference
Marriage status				
Married	0.75(0.69 ~ 0.81) ^***^	0.81(0.71 ~ 0.93) ^**^	0.73(0.65 ~ 0.82) ^***^	0.91(0.75 ~ 1.10)
Unmarried	Reference	Reference	Reference	Reference
HIV status				
Positive	2.12(1.80 ~ 2.49) ^***^	1.70(1.23 ~ 2.35) ^**^	5.22(4.44 ~ 6.15) ^***^	4.73(3.53 ~ 6.35) ^***^
Negative	Reference	Reference	Reference	Reference
HCV status				
Positive	1.87(1.70 ~ 2.06) ^***^	2.58(2.19 ~ 3.04) ^***^	1.67(1.40 ~ 1.99) ^***^	1.97(1.51 ~ 2.58) ^***^
Negative	Reference	Reference	Reference	Reference
MMT mean dosage	1.00^a^(1.00^b^ ~ 1.00^c^) ^*^	1.00^d^(0.99 ~ 1.00^e^) ^**^	1.00(1.00 ~ 1.00)	1.00(1.00 ~ 1.00)
Treatment adherence	0.42(0.36 ~ 0.48) ^***^	0.18(0.16 ~ 0.19) ^***^	0.34(0.30 ~ 0.38) ^***^	0.42(0.36 ~ 0.48) ^***^
Longest duration of abstinence	0.86(0.85 ~ 0.87) ^***^	0.91(0.89 ~ 0.93) ^***^	0.86(0.84 ~ 0.88) ^***^	0.92(0.89 ~ 0.95) ^***^
Unprotected sex				
Yes	1.05(0.95 ~ 1.16)	1.13(0.99 ~ 1.29)	1.13(0.96 ~ 1.33)	1.27(1.06 ~ 1.53) ^***^
No	Reference	Reference	Reference	Reference
Multiple sexual partners				
Yes	1.11(0.96 ~ 1.29)	0.91(0.75 ~ 1.12)	1.42(1.15 ~ 1.77) ^**^	1.49(1.17 ~ 1.90) ^**^
No	Reference	Reference	Reference	Reference

^*^P < 0.05, ^**^P < 0.01, ^***^P < 0.001.

Exact value: ^a^: 0.998; ^b^: 0.996; ^c^: 0.999; ^d^: 0.995; ^e^: 0.998.

**Table 5:** Generalized linear mixed models (GLMM) logistic regression results: Predictors of continued sexual risk behaviors

**Variables**	**Unprotected sex**		**Multiple sexual partners**	
	**Unadjusted OR**	**Adjusted OR**	**Unadjusted OR**	**Adjusted OR**
Age	1.03(1.03 ~ 1.04) ^***^	1.02(1.01 ~ 1.03) ^***^	0.97(0.96 ~ 0.98) ^***^	0.98(0.97 ~ 1.00^a^) ^*^
Years of drug use before MMT	1.02(1.01 ~ 1.03) ^***^	1.01(1.00^b^ ~ 1.02) ^*^	0.99(0.97 ~ 1.00)	0.99(0.98 ~ 1.01)
Gender				
Male	1.03(0.86 ~ 1.22)	1.10(0.92 ~ 1.32)	0.80(0.60 ~ 1.08)	0.80(0.59 ~ 1.08)
Female	Reference	Reference	Reference	Reference
Employment status				
Employed	0.80(0.72 ~ 0.88) ^***^	0.82(0.74 ~ 0.91) ^***^	1.09(0.93 ~ 1.27)	1.16(0.99 ~ 1.36)
Unemployed	Reference	Reference	Reference	Reference
Education				
Senior school or higher	1.08(0.92 ~ 1.26)	1.11(0.94 ~ 1.31)	0.94(0.73 ~ 1.22)	0.98(0.75 ~ 1.27)
Junior school	0.99(0.87 ~ 1.12)	1.02(0.89 ~ 1.17)	1.16(0.95 ~ 1.42)	1.11(0.90 ~ 1.36)
Primary school	Reference	Reference	Reference	Reference
Marriage status				
Married	1.43(1.30 ~ 1.58) ^***^	1.35(1.21 ~ 1.50) ^***^	0.57(0.49 ~ 0.67) ^***^	0.58(0.49 ~ 0.69) ^***^
Unmarried	Reference	Reference	Reference	Reference
HIV status				
Positive	0.43(0.34 ~ 0.55) ^***^	0.43(0.33 ~ 0.55) ^***^	0.61(0.42 ~ 0.89) ^*^	0.49 (0.33 ~ 0.74) ^**^
Negative	Reference	Reference	Reference	Reference
HCV status				
Positive	0.97(0.85 ~ 1.11)	0.84(0.73 ~ 0.96) ^***^	1.48(1.21 ~ 1.83) ^***^	1.47(1.18 ~ 1.84) ^**^
Negative	Reference	Reference	Reference	Reference
MMT mean dosage	0.99(0.99 ~ 1.00^c^) ^***^	1.00^d^(0.99 ~ 1.00^e^) ^***^	1.00(1.00 ~ 1.00)	1.00(1.00 ~ 1.01)
Treatment adherence	1.00^f^(0.99 ~ 1.00^g^) ^*^	0.99(0.99 ~ 1.00^h^) ^***^	0.98(0.97 ~ 0.99) ^***^	0.98(0.97 ~ 0.99) ^***^
Longest duration of abstinence	0.99(0.97 ~ 1.00^i^) ^*^	0.96(0.94 ~ 0.98) ^***^	0.97(0.95 ~ 0.99) ^**^	0.97 (0.95 ~ 1.00^j^) ^*^
Drug injection				
Yes	1.12(0.99 ~ 1.27)	0.75(0.65 ~ 0.86)^***^	0.92(0.75 ~ 1.13)	0.93(0.74 ~ 1.15)
No	Reference	Reference	Reference	Reference
Syringes sharing				
Yes	0.88(0.77 ~ 1.00)	0.97(0.84 ~ 1.12)	0.77(0.63 ~ 0.93) ^**^	0.77(0.63 ~ 0.95) ^*^
No	Reference	Reference	Reference	Reference

^*^P < 0.05, ^**^P < 0.01, ^***^P < 0.001.

Exact value: ^a^: 0.999; ^b^: 1.001 ^c^: 0. 996; ^d^: 0.996; ^e^: 0.998; ^f^: 0.996; ^g^: 0.999; ^h^: 0.997; ^i^: 0.999; ^j^: 0.996.

The following variables significantly increased the risk of sharing needles: younger age, longer history of drug use, being HIV or HCV positive, lower level of treatment adherence, shorter duration of abstinence, having unprotected sex prior to MMT and having multiple sexual partners prior to MMT.

The significant predictors for unprotected sex included younger age, longer history of drug use, unemployment, being married, being HIV or HCV positive, lower mean dosage of methadone, lower level of treatment adherence, shorter duration of abstinence and injecting drugs prior to MMT.

The significant predictors for multiple sexual partners included younger age, being unmarried, HIV negative, HCV positive, lower level of treatment adherence, shorter duration of abstinence and sharing needles prior to MMT.

## Discussion

Our data revealed that MMT client HIV-risk behaviors had decreased over the course of treatment, especially in the first 6 months, indicating positive effects of MMT on HIV prevention. These findings were consistent with previous studies on the effect of MMT [9,39-41], especially in China [42,43]. We found that HIV-positive clients were 1.70 times more likely to continue to engage in drug injection and 4.73 times more likely to continue to share syringes compared to their counterparts at 12 month follow-up, even after controlling for other factors (Table 4), suggesting that awareness of HIV status and HIV counseling may not be sufficient to reduce drug-related risk behaviors [44-46]. In contrast to their drug-related behaviors, HIV-positive individuals in our study were less likely to continue to have unprotected sex and have multiple sexual partners (Table 5). Although HIV-prevention counseling is a part of MMT programs in China, our data underscore the inadequacy or limited effect of drug-related HIV-prevention counseling. Programs to reduce drug-risk behaviors targeting HIV-positive individuals are urgently needed [40,47,48].

HCV-positive clients were 2.58 times more likely to continue to inject drugs, 1.97 times more likely to continue to share syringes (Table 4), and 1.47 times more likely to continue to have multiple sexual partners (Table 5); their drug-related HIV-risk behaviors resembled those in previous studies [49-51]. More than 70% of IDU in Guangdong province are infected with HCV (in our study 78% were HCV positive) [52], but HCV counseling and treatment were not part of the MMT protocol partly because the MMT programs were established in response to the growing HIV/AIDS epidemic. Our data suggest an urgent need to include effective HCV-prevention counseling in MMT.

We note that the most significant factor in reducing HIV risks was treatment adherence; in other words, MMT clients who better adhered to the treatment and stayed in the program longer were more likely to reduce their drug- or sex-related risk behaviors, suggesting that more exposure to MMT was effective in reducing HIV risks. Such findings suggest the need for measures to promote adherence among MMT clients. Inconsistent results were reported in previous studies on the relationship between treatment adherence and methadone dosage. Ramli M et al. demonstrated that there was a positive association between these two factors [53], on the contrary, methadone maintenance dose was not associated with improved treatment adherence in Zhao L’s study [54]. The current study did not focus on this relationship; however, further studies are clearly required to investigate impact of methadone dose on treatment adherence. Other risk factors included younger age, longer history of drug abuse, being male, unemployment, less education, and being single. These findings provide important information by identifying at-risk individuals who could benefit from targeted interventions to reduce their HIV risk.

The current study has several limitations. First, we had a relatively high attrition rate: 38% at first follow-up and 61% at second follow-up. We did not employ any retention efforts during the study as it was a longitudinal observational study rather than an intervention study. Although the rate of dropout is high, this is typical in MMT clinics and highlights the need for future research to increase retention in these programs. The attrition rates observed in the current study were similar to previous MMT studies in China [20-23]. Nevertheless, the data presented in the current study might be biased towards “good clients.” We learned that those who dropped out were more likely to be male, younger, unemployed, and HIV positive and have shorter history of drug abuse. Therefore, future studies are needed to target these drug users who were more likely to drop out of MMT and determine reasons for dropping out. Second, we have used self-reported data for all study predictors; thus, recall or social desirability biases might be present. Third, we included only a limited number of outcome and independent variables in the survey; other important psychosocial measures, such as mental health status as well as structural factors such as social support and social networks [55] were not included. These variables might explain more about the relationship between continued HIV-risk behaviors and MMT characteristics. Finally, our study was conducted in selected MMT clinics in Guangdong Province of south China; the data might not be generalizable to other MMT clients living elsewhere in China.

Despite these limitations, our study represents one of the first efforts to use a prospective design to evaluate the factors associated with continued HIV-risk behaviors during the course of MMT. We found that HIV- and HCV-positive status were strongly associated with drug- and sex-related risk behaviors, which underscore the urgency to strengthen HIV counseling, especially drug-risk reduction in the current MMT programs. Given the high prevalence of HCV in China we adding counseling services for HCV- positive MMT clients is urgently needed. The high attrition rate and strong association of MMT adherence and HIV-risk reduction suggest the importance of enhancing adherence among MMT clients. MMT has been scaled-up nationwide in China since 2006 [17], and its “revolving-door” effect has been reported in previous studies [10,56,57]. Because the positive effect of MMT and its extensive reach have been confirmed in the past decade [2,5,6], now is a critical time to adjust the program to improve implementation effectiveness. Motivating drug users to adhere to the program longer and reducing HIV risks and drug abuse require greater efforts than the MMT program. They require structural interventions, such as programs to reduce the stigma associated with drug users and HIV-positive individuals as well as a social support system for vulnerable drug users, especially younger, unmarried, and unemployed individuals.

## Conclusions

Our data confirmed the positive effects of MMT on HIV prevention and underscored the urgency for programs to reduce HIV risk in HIV- and HCV-positive clients. We call to strengthen the existing counseling services for HIV-positive drug users to reduce their drug-related risk behaviors and to provide counseling for HCV-positive drug users. Future studies are needed to explore interventions to address high dropout rates and low adherence among MMT clients.

## Competing interests

All authors have declared no conflicts of interest.

## Authors’ contributions

LL conceptualized the study and wrote the protocol, WC and XY led the data collection and, WC led the data analysis and wrote the first draft of the manuscript. YH led the manuscript development. BJH contributed to the writing and preparation of the manuscript revision. All authors have contributed to and approved the final manuscript.
